# Health and wellbeing among the empty nest and non-empty nest elderly in China—Results from a national cross-sectional study

**DOI:** 10.1371/journal.pone.0291231

**Published:** 2023-09-12

**Authors:** Sijie Xu, Xiaocong Yang, Jieyu Liu, Marc Ka-chun Chong, Yu Cheng, Weiwei Gong, Guanyang Zou

**Affiliations:** 1 School of Public Health and Management, Guangzhou University of Chinese Medicine, Guangzhou, China; 2 School of Public Management, Guangzhou University, Guangzhou, China; 3 SOAS China Institute, School of Oriental and African Studies, University of London, London, England; 4 The Jockey Club School of Public Health and Primary Care, Chinese University of Hong Kong, Hong Kong, China; 5 School of Social Science and Anthropology, Sun Yat-sen University, Guangzhou, China; 6 Zhejiang Provincial Center for Disease Control and Prevention, Hangzhou, China; University of Bergen: Universitetet i Bergen, NORWAY

## Abstract

**Background:**

The number of empty nest elderly in China has gradually increased in recent years. There is growing concern about the physical and mental health of this population as empty nest elderly are commonly at the risk of compromising health, home safety and quality of life. This study reported the health and well-being of empty nest elderly with regards to their health status, depression and satisfaction, lifestyle as compared to non-empty nest elderly in China.

**Methods:**

Data was collected from the 2018 follow-up interviews of China Health and Retirement Longitudinal Survey. We included 4,630 empty nest elderly and 6,188 non-empty nest elderly. Chi-square Test and Logistic Regression were used to compare the differences between these two groups.

**Results:**

As compared to the non-empty nest elderly, there was higher proportion of empty nest elderly who suffered from dyslipidemia, diabetes, chronic lung diseases, heart attack (27.0% vs. 25.0%; 16.6% vs. 15.1%; 19.4% vs. 16.4%; 26.3% vs. 23.4%, P < 0.05). The empty nest elderly had higher proportion of participants who drank more than once a month (25.3% vs. 23.9%, P < 0.05), who felt satisfied with their marriage (71.6% vs. 66.2%, P < 0.001), who were satisfied with their children’s relationship (85.2% vs. 83.2%, P < 0.001). However, these significances disappeared in the Logistic Regression analysis (P > 0.05).

**Conclusion:**

Our study showed that significant between-group difference was found between empty nest elderly and non-empty nest elderly in their health and wellbeing. However, disappearance of such difference in the multivariable analysis may indicate improved health and wellbeing among the empty nest elderly. Even though our study still suggested the importance of improving the health, lifestyles and family dynamics of the elderly and promoting the integration of health and social care for the elderly, especially among the empty nest elderly.

## Introduction

China is almost a moderately aging society, with 18.7% of the total population above 60 years old [1]. With technological developments (e.g. in transport and communication), urbanization, migration and changing gender norms [2], the number of empty nest elderly in China has gradually increased in recent years- with nearly half of the elderly being empty nest elderly [3]. It is estimated that by 2030, the number of empty nest elderly households will account for 90% of elderly households [4]. There is growing concern about the physical and mental health of this population as empty nest elderly are commonly at the risk of compromising health, home safety and quality of life. This has caused increasing social and economic burden due to the lack of care and company from their children [5].

Studies have shown that empty nest elderly and non-empty nest elderly had significant differences in many aspects such as physical and mental health, social support. Nayak et al. [6] found that empty nesting was associated with depressive symptoms in older adults and depression was more serious among older people in empty nest elderly without partners in India. In Mexico, Antman [7] suggested that empty nest aging parents were associated with poorer physical and mental health outcomes. Randall Kuhn et al. [8] found that empty nesting had a negative impact on activities of daily living (ADL), self-rated health (SRH) and mortality of elderly parents. Previous studies in China showed that, compared with non-empty nest elderly, empty nest elderly had poorer physical health [9], lower subjective well-being [10], poorer cognitive ability [9], poorer mental health [9,11], higher loneliness [12], higher degree of depression [13], lower life satisfaction [14], lower quality of life related to health [15] and poorer social relations [14,16]. However, other studies from China have not found significant differences between these two elderly groups, for example, in terms of loneliness and cognition [17].

To date, many of the previous studies in China have focused on one dimension of health and focused on one region and have a lack of comparison between empty and non-empty nest elderly. Using the national survey data, this study aims to compare the health and well-being between empty nest elderly and non-empty nest elderly, with regards to the demographic factors, health status, depression, satisfaction and lifestyle. Our study will provide reference for promoting healthy aging and improving the welfare, instrumental and emotional support from adult children especially among empty nest elderly.

## Methods

### Data source and study sample

**Fig 1:**
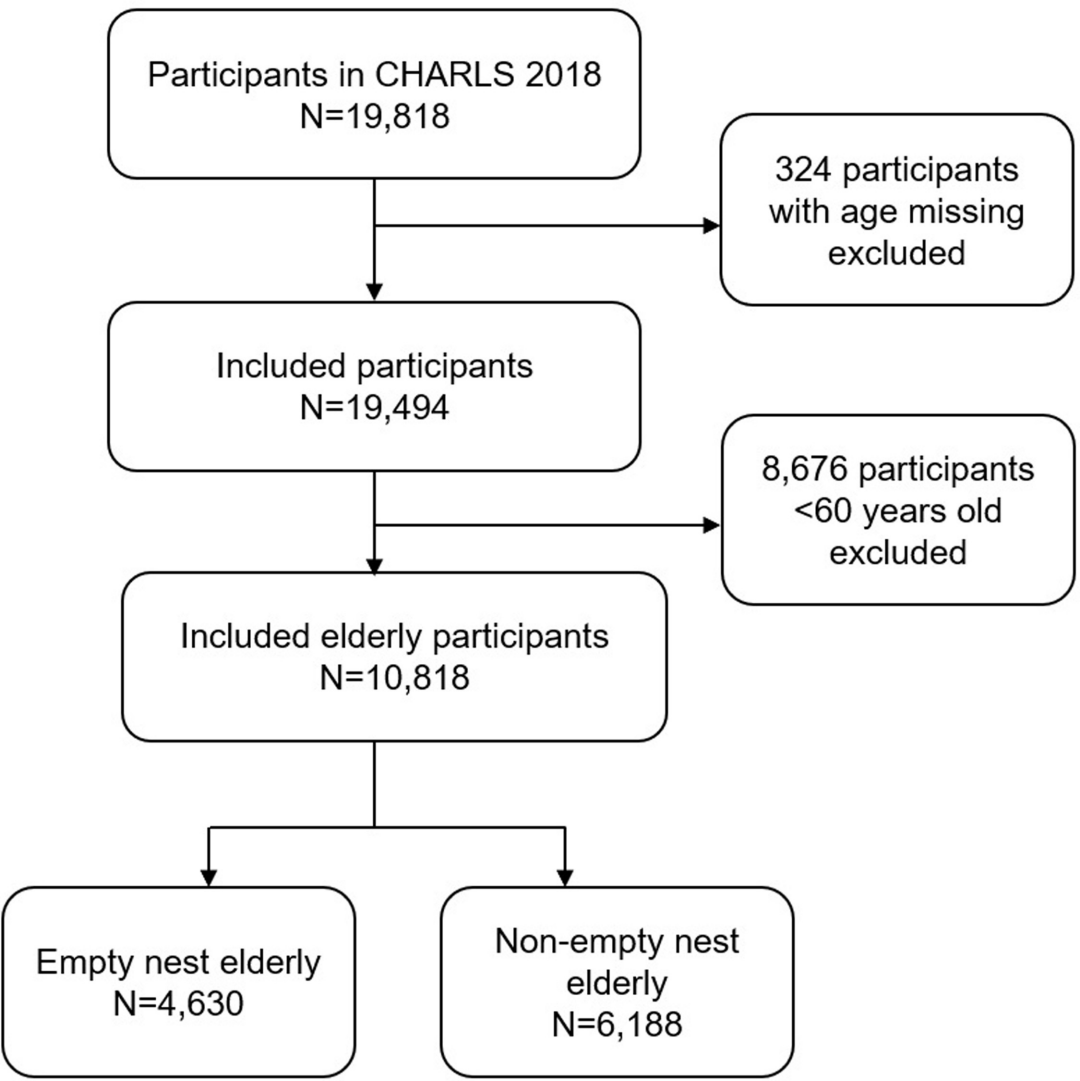
Flow chart of study sample selection. Fig 1 Flow chart of the selected empty nest elderly and non-empty nest elderly for our study in China Health and Retirement Longitudinal Survey in 2018 (CHARLS2018).

The data used in this paper was exported from the national survey of China Health and Retirement Longitudinal Survey in 2018 (CHARLS2018). CHARLS is a nationwide, population-based cohort study, designed and implemented by the National Development Research Institute of Peking University since January 1, 2011. It examines the health and economic situation of the rapidly aging population in China [18]. The baseline survey was conducted in 28 provinces, 150 countries/ districts, 450 villages/ urban communities across the country in 2011–2012, with wave 2, wave 3 and wave 4 conducted in 2013, 2015 and 2018 respectively. CHARLS2018 is the latest national representative survey data of middle-aged and elderly people. Using a stratified multi-stage probability proportional to size random sampling strategy, CHARLS2018 collected data from 19,818 participants [19]. The CHARLS 2018 database consists of several sub-databases, and the sub-databases involved in this study were the Demographic, Family Information, Family Transfer, Cognition, Health Status and Functioning. We created separate weights for households, individuals, and biomarkers to deal with nonresponse bias and sampling-frame errors. After excluding participants with age missing (n = 324) and those <60 years (n = 8676), finally, a total of 10,818 valid samples were included in our study, covering 4,630 empty nest elderly and 6,188 non-empty nest elderly (Fig 1).

Ethical approval for all the CHARLS waves was granted from the Institutional Review Board at Peking University. The IRB approval number for the main household survey, including anthropometrics, was IRB00001052-11015; the IRB approval number for biomarker collection, was IRB00001052-11014. During the fieldwork, each respondent who agreed to participate in the survey was asked to sign two copies of the informed consent. Authors had no access to information that could identify individual participants during and after data collection. This study followed the Strengthening the Reporting of Observational Studies in Epidemiology (STROBE) reporting guideline.

### Key variable definitions

**Empty nest elderly.** According to previous studies, empty nest elderly are defined as those aged 60 years or older, having no children or living alone without their children for 12 months [16,20–25]. It is a self-reported living arrangement mainly based on the questions of “Where does [XChildName[i]] live?” “During last year, how long had [XChildName[i]] lived with you and your spouse?”, regardless the consideration of physical distance and time duration with home leavers. In our study, these elderly people either lived alone without their spouses (empty nest singles) or with their spouses (empty nest couples). They might also be carer(s) of their parents, live with their grandchildren, live with other family relatives, so the empty nest family does not really mean “living without other family members”.

### Variables

The variable of empty nesting or not was obtained by reconstructing the “Number of Children” variable and combining it with the “Where is [XChildName[i]] Living Regularly” question and the “How Long Live with XChildName[i]” question ‐ where the number of children for non-empty nest elderly was not “0”; in the “Where is [XChildName[i]] Living Regularly” question, those who showed “living with family respondent” were non-empty nest elderly, otherwise were empty nest elderly; in the “How Long Live with XChildName[i]” question, those who showed “0 (months)” were empty nest elderly, otherwise were non-empty nest elderly. The socio-demographic variables included age, gender, number of children, type of residence, location of residential address, education and marital status. Health status variables included self-assessed health status, diagnosed with hypertension, diagnosed with dyslipidemia, diagnosed with diabetes, diagnosed with chronic lung diseases, diagnosed with heart attack, diagnosed with stroke, diagnosed with emotional, nervous or psychiatric problems. Depression and satisfaction variables included have depressive symptoms, life satisfaction, health satisfaction, marital satisfaction and satisfaction with children’s relationship. Depression is measured based on the CES-D10 scale, which covers 10 questions in the CHARLS2018 questionnaire. Participants with total score ≥10 and <10 are judged as having or not having depressive symptoms. Lifestyle variables included average sleep, vigorous-intensity physical activities, moderate physical activities, mild physical activities, social activities, frequency of drinking.

### Statistical analysis

Stata 17.0 statistic software was used to conduct statistical analysis (See S1 File for the selected data set). Descriptive analysis was used to report the socio-demographic information, health status, depression and satisfaction, lifestyle of empty nest elderly and non-empty nest elderly. Chi-square test was used to analyze and compare the differences between these two groups in these variables. Binomial Logistic Regression and Multinominal Logistic Regression were employed to confirm whether the status of empty nesting was associated with the health status, depression, satisfaction, lifestyle and other variables of the elderly. Significant variables (P < 0.05) derived from the between-group analysis were included in the logistic regression. The socio-demographic variables (including number of children, age, gender, type of residential address, location of residential address, highest level of education and marital status variables) were used as control variables in logistic regression analysis. P < 0.05 was considered statistically significant.

## Results

### Demographic comparison of empty nest elderly and non-empty nest elderly population

Of 10,818 elderly people, 4,630 (42.8%) and 6,188 (57.2%) were empty nest elderly and non-empty nest elderly respectively. As compared to the non-empty nest elderly, empty nest elderly had significantly higher proportion of men (51.9%vs. 48.1%, P < 0.05), those who were "married and living with spouse” (78.4% vs. 70.7%, P < 0.001), had lower proportion of participants who were aged≥ 80 (13.6% vs. 13.9%, P < 0.001), who had a number of children ≥4 (0.0% vs. 1.3%, P < 0.001), who lived in the family house (97.5% vs. 98.9%, P < 0.001). No statistical difference was found between empty nest elderly and non-empty nest elderly regarding residential address location (P = 0.386) and education (P = 0.683) (Table 1 and see S2 File for the weighted process).

**Table 1:** Demographic sociological comparison of non-empty nest elderly and empty nest elderly.

	Non-empty Nest Elderly		Empty Nest Elderly		Total		p-value
	N = 6188		N = 4630		N = 10818		
**Age**							<0.001***
≥60, <70	3856	(59.30%)	2616	(53.70%)	6472	(56.90%)	
≥70, <80	1690	(26.80%)	1529	(32.70%)	3219	(29.30%)	
≥80	642	(13.90%)	485	(13.60%)	1127	(13.70%)	
**Gender**							0.002**
Female	3237	(51.90%)	2275	(48.10%)	5512	(50.30%)	
Male	2951	(48.10%)	2355	(51.90%)	5306	(49.70%)	
**Number of Children**							<0.001***
0	0	(0.00%)	4610	(99.70%)	4610	(41.90%)	
1	5078	(82.50%)	11	(0.20%)	5089	(47.90%)	
2	837	(13.40%)	2	(0.00%)	839	(7.80%)	
3	181	(2.70%)	5	(0.00%)	186	(1.60%)	
≥4	92	(1.30%)	2	(0.00%)	94	(0.80%)	
**Location of Residential Address**							0.386
City	1086	(24.30%)	876	(27.10%)	1962	(25.40%)	
Village	4692	(67.90%)	3418	(64.80%)	8110	(66.60%)	
Other areas	392	(7.60%)	299	(7.50%)	691	(7.50%)	
Missing	18	(0.20%)	37	(0.70%)	55	(0.40%)	
**Type of Residential Address**							<0.001***
Nursing home, hospital, other	59	(0.80%)	102	(1.80%)	161	(1.30%)	
Family housing	6111	(98.90%)	4491	(97.50%)	10602	(98.30%)	
Missing	18	(0.20%)	37	(0.70%)	55	(0.40%)	
**Education**							0.683
Illiterate	1925	(29.20%)	1389	(27.20%)	3314	(28.40%)	
Primary and middle school	3681	(58.90%)	2788	(60.70%)	6469	(59.70%)	
High school and above	582	(11.90%)	453	(12.10%)	1035	(12.00%)	
**Marital Status**							<0.001***
Married and living with spouse	4450	(70.70%)	3649	(78.40%)	8099	(74.00%)	
Married but don’t living with spouse, separated, divorced, widowed or never married	1738	(29.30%)	981	(21.60%)	2719	(26.00%)	

Source: CHARLS 2018.

Notes: 1) the number of respondents and weighted percentage of the sample (in parentheses) are presented; 2) the chi-square test has been used to show the between-groups-difference, and Sig:

* p<0.05

** p<0.01

*** p<0.001.

### Comparison of health status between empty nest elderly and non-empty nest elderly

As compared to non-empty nest elderly, empty nest elderly had higher proportion of participants with dyslipidemia (27.0% vs. 25.0%, P < 0.05), diabetes (16.6% vs. 15.1%, P < 0.05), chronic lung diseases (19.4% vs. 16.4%, P < 0.05) and heart attack (26.3% vs. 23.4%, P < 0.05). No statistical difference was found between empty nest elderly and non-empty nest elderly regarding self-assessed health status (P = 0.318), whether being diagnosed with hypertension (P = 0.676), stroke (P = 0.867) and emotional problems (P = 0.152) (Table 2 and see S3 File for the weighted process).

**Table 2:** Comparison of health status between non-empty nest elderly and empty nest elderly.

	Non-empty Nest Elderly		Empty Nest Elderly		Total		p-value
	N = 6188		N = 4630		N = 10818		
**Self-reported Health**							0.318
Poor or worst	1729	(26.20%)	1325	(26.40%)	3054	(26.30%)	
Fair	2655	(43.70%)	2097	(46.30%)	4752	(44.80%)	
Good	1228	(19.80%)	864	(19.40%)	2092	(19.60%)	
Missing	576	(10.30%)	344	(7.90%)	920	(9.30%)	
**Hypertension**							0.676
No	3314	(53.20%)	2486	(51.90%)	5800	(52.60%)	
Yes	2864	(46.70%)	2114	(47.30%)	4978	(46.90%)	
Missing	10	(0.20%)	30	(0.80%)	40	(0.50%)	
**Dyslipidemia**							0.006**
No	4725	(74.80%)	3398	(72.10%)	8123	(73.70%)	
Yes	1453	(25.00%)	1202	(27.00%)	2655	(25.90%)	
Missing	10	(0.20%)	30	(0.80%)	40	(0.50%)	
**Diabetes**							0.003**
No	5281	(84.70%)	3840	(82.60%)	9121	(83.80%)	
Yes	896	(15.10%)	760	(16.60%)	1656	(15.70%)	
Missing	11	(0.20%)	30	(0.80%)	41	(0.50%)	
**Chronic Lung Diseases**							0.011*
No	5117	(83.40%)	3712	(79.80%)	8829	(81.90%)	
Yes	1060	(16.40%)	888	(19.40%)	1948	(17.60%)	
Missing	11	(0.20%)	30	(0.80%)	41	(0.50%)	
**Heart Attack**							0.001**
No	4725	(76.40%)	3383	(72.90%)	8108	(74.90%)	
Yes	1452	(23.40%)	1217	(26.30%)	2669	(24.60%)	
Missing	11	(0.20%)	30	(0.80%)	41	(0.50%)	
**Stroke**							0.867
No	5561	(89.10%)	4138	(89.10%)	9699	(89.10%)	
Yes	616	(10.70%)	462	(10.00%)	1078	(10.40%)	
Missing	11	(0.20%)	30	(0.80%)	41	(0.50%)	
**Emotional Problems**							0.152
No	6009	(97.20%)	4446	(96.20%)	10455	(96.80%)	
Yes	168	(2.60%)	153	(2.90%)	321	(2.70%)	
Missing	11	(0.20%)	31	(0.80%)	42	(0.50%)	

Source: CHARLS 2018.

Notes: 1) the number of respondents and weighted percentage of the sample (in parentheses) are presented; 2) the chi-square test has been used to show the between-groups-difference, and Sig:

* p<0.05

** p<0.01

*** p<0.001.

### Comparison of depression and satisfaction between empty nest elderly and non-empty nest elderly

As compared to non-empty nest elderly, empty nest elderly had higher proportion of participants who felt satisfied with their marriage (71.6% vs. 66.2%, P < 0.001), who were satisfied with their children’s relationship (85.2% vs. 83.2%, P < 0.001). No statistical difference was found between empty nest elderly and non-empty nest elderly regarding the presence of depressive symptoms (P = 0.553), life satisfaction (P = 0.573) and health satisfaction (P = 0.297) (Table 3 and see S4 File for the weighted process).

**Table 3:** Comparison of depression and satisfaction between non-empty nest elderly and empty nest elderly.

	Non-empty Nest Elderly		Empty Nest Elderly		Total		p-value
	N = 6188		N = 4630		N = 10818		
**CESD-10**							0.553
No Depressive Symptoms	4094	(68.10%)	3041	(67.90%)	7135	(68.00%)	
Have Depressive Symptoms	2094	(31.90%)	1589	(32.10%)	3683	(32.00%)	
**Life Satisfaction**							0.573
Not satisfied	615	(9.40%)	452	(9.20%)	1067	(9.30%)	
Satisfied	4929	(79.20%)	3814	(82.50%)	8743	(80.60%)	
Missing	644	(11.40%)	364	(8.30%)	1008	(10.10%)	
**Health Satisfaction**							0.297
Not satisfied	1531	(24.60%)	1208	(25.50%)	2739	(25.00%)	
Satisfied	4013	(64.00%)	3058	(66.10%)	7071	(64.90%)	
Missing	644	(11.40%)	364	(8.30%)	1008	(10.10%)	
**Marriage Satisfaction**							<0.001***
Satisfied	4202	(66.20%)	3344	(71.60%)	7546	(68.50%)	
Not satisfied	464	(7.70%)	376	(7.70%)	840	(7.70%)	
No Spouse	878	(14.70%)	546	(12.40%)	1424	(13.70%)	
Missing	644	(11.40%)	364	(8.30%)	1008	(10.10%)	
**Children Satisfaction**							<0.001***
Satisfied	5248	(83.20%)	3964	(85.20%)	9212	(84.00%)	
Not satisfied	292	(5.40%)	208	(4.30%)	500	(4.90%)	
No Child	4	(0.10%)	94	(2.20%)	98	(1.00%)	
Missing	644	(11.40%)	364	(8.30%)	1008	(10.10%)	

Source: CHARLS 2018.

Notes: 1) the number of respondents and weighted percentage of the sample (in parentheses) are presented; 2) the chi-square test has been used to show the between-groups-difference, and Sig:

* p<0.05

** p<0.01

*** p<0.001.

### Comparison of lifestyle between empty nest elderly and non-empty nest elderly

As compared to the non-empty nest elderly, empty nest elderly had higher proportion of participants who drank more than once a month (25.3% vs. 23.9%, P < 0.05). No statistical difference was found between empty nest elderly and non-empty nest elderly regarding average sleeping time per night (P = 0.970), whether intensive physical activities exceeding 10 minutes each time (P = 0.742), whether moderate physical activities exceeding 10 minutes each time (P = 0.157), whether light physical activities exceeding 10 minutes each time (P = 0.597) and whether participating in social activities (P = 0.882) (Table 4 and see S5 File for the weighted process).

**Table 4:** Comparison of lifestyle between non-empty nest elderly and empty nest elderly.

	Non-empty Nest Elderly		Empty Nest Elderly		Total		p-value
	N = 6188		N = 4630		N = 10818		
**Average Sleep Time**							0.970
≤5	2229	(35.00%)	1655	(35.40%)	3884	(35.10%)	
>5, ≤7	2138	(36.30%)	1619	(36.00%)	3757	(36.20%)	
>7	1821	(28.80%)	1356	(28.60%)	3177	(28.70%)	
**Intensive Physical Activity More than 10 Mins Each Time**							0.742
No	4574	(76.10%)	3419	(76.60%)	7993	(76.30%)	
Yes	1601	(23.60%)	1178	(22.50%)	2779	(23.20%)	
Missing	13	(0.20%)	33	(0.90%)	46	(0.50%)	
**Moderate Physical Activity More than 10 Mins Each Time**							0.157
No	3503	(57.20%)	2663	(56.90%)	6166	(57.10%)	
Yes	2672	(42.50%)	1934	(42.30%)	4606	(42.40%)	
Missing	13	(0.20%)	33	(0.90%)	46	(0.50%)	
**Light Physical Activity More than 10 Mins Each Time**							0.597
No	1229	(20.00%)	885	(18.10%)	2114	(19.20%)	
Yes	4946	(79.70%)	3712	(81.00%)	8658	(80.30%)	
Missing	13	(0.20%)	33	(0.90%)	46	(0.50%)	
**Social activities in Last Month**							0.882
No	3238	(50.90%)	2414	(49.20%)	5652	(50.20%)	
Yes	2936	(48.80%)	2183	(49.90%)	5119	(49.30%)	
Missing	14	(0.20%)	33	(0.90%)	47	(0.50%)	
**Frequency Drank Alcoholic Beverages in the Past Year**							0.037*
None of These	4309	(68.80%)	3111	(66.60%)	7420	(67.90%)	
Drink but less than once a month	417	(7.10%)	308	(7.20%)	725	(7.10%)	
Drink more than once a month	1448	(23.90%)	1177	(25.30%)	2625	(24.50%)	
Missing	14	(0.20%)	34	(0.90%)	48	(0.50%)	

Source: CHARLS 2018.

Notes: 1) the number of respondents and weighted percentage of the sample (in parentheses) are presented; 2) the chi-square test has been used to show the between-groups-difference, and Sig:

* p<0.05

** p<0.01

*** p<0.001.

### Influence of "empty nesting” on health status and other variables: Based on multivariable analysis

The Binomial Logistic Regression Analysis did not show significant difference between the empty nest elderly and non-empty nest elderly regarding dyslipidemia, diabetes, chronic lung diseases and heart attack (P > 0.05). Similarly, based on the Multinomial Logistic Regression Analysis, no significant difference was found between these two groups regarding their satisfaction with relationships with spouses and children and frequency of drinking alcohol (P > 0.05) (Table 5).

**Table 5:** Regression analysis for full sample.

	(1)	(2)	(3)	(4)	(5)		(6)		(7)	
	Dyslipidemia	Diabetes	Chronic lung diseases	Heart attack	Marriage Satisfaction		Children satisfaction		Frequency Drank Alcoholic	
					Not satisfied	Satisfied	Not satisfied	Satisfied	Less than Once/Month	More than Once/Month
main										
Whether Empty Nest Elderly	0.345	0.789	0.892	-1.258	-1.887	-2.553	14.207	13.082	-13.088	-1.294
	(0.772)	(0.844)	(0.967)	(1.084)	(2.651)	(2.488)	(5729.690)	(5729.690)	(620.520)	(1.099)
Age groups (Refs: ≥60 & <70)										
Age ≥70 & <80	0.029	0.080	0.239***	0.438***	-0.542***	-0.574***	0.578**	0.539**	-0.196**	-0.332***
	(0.066)	(0.077)	(0.071)	(0.064)	(0.118)	(0.097)	(0.272)	(0.254)	(0.090)	(0.057)
Age >80	-0.320***	-0.115	0.431***	0.373***	-0.500***	-0.740***	0.772**	0.699*	-0.334**	-0.699***
	(0.112)	(0.130)	(0.109)	(0.100)	(0.176)	(0.132)	(0.393)	(0.362)	(0.146)	(0.096)
Gender (Refs: Female)	-0.381***	-0.250***	0.219***	-0.518***	-0.588***	0.560***	-2.368***	-2.301***	0.876***	2.206***
	(0.069)	(0.080)	(0.076)	(0.066)	(0.123)	(0.099)	(0.321)	(0.307)	(0.086)	(0.063)
Number of Children (Refs: None)										
1	0.266	0.712	0.674	-1.366	-1.807	-2.417	17.533	16.395	-13.109	-1.384
	(0.773)	(0.845)	(0.967)	(1.084)	(2.652)	(2.489)	(5729.690)	(5729.690)	(620.520)	(1.100)
2	0.138	0.419	0.647	-1.753	-1.898	-2.335	30.136	29.028	-13.063	-1.317
	(0.791)	(0.860)	(0.975)	(1.092)	(2.658)	(2.493)	(5773.566)	(5773.566)	(620.520)	(1.102)
3	-0.010	0.497	0.848	-1.232	-0.952	-2.075	30.881	29.221	-12.867	-1.598
	(0.803)	(0.882)	(0.993)	(1.102)	(2.668)	(2.504)	(5934.511)	(5934.511)	(620.520)	(1.110)
≥4	0.187	0.907	1.079	-1.000	-1.933	-2.363	30.194	29.387	-12.886	-1.844
	(0.809)	(0.875)	(0.990)	(1.102)	(2.703)	(2.517)	(6164.937)	(6164.937)	(620.520)	(1.139)
Location (Refs: City)										
Village	-0.600***	-0.384***	0.252**	-0.440***	0.535***	0.163	0.408	0.323	-0.315***	-0.004
	(0.081)	(0.095)	(0.110)	(0.081)	(0.156)	(0.125)	(0.338)	(0.313)	(0.101)	(0.070)
Other areas	0.294**	0.256	0.510***	0.295**	0.471*	0.314	-0.228	-0.617	0.106	0.214*
	(0.135)	(0.168)	(0.164)	(0.139)	(0.260)	(0.213)	(0.486)	(0.449)	(0.161)	(0.115)
Family Housing (Refs: Nursing Home, etc)	0.553**	0.171	0.468*	0.084	-0.930**	-0.712**	-0.339	0.254	0.019	0.071
	(0.246)	(0.273)	(0.258)	(0.211)	(0.380)	(0.291)	(0.542)	(0.474)	(0.305)	(0.197)
Education (Refs: Illiterate)										
Primary and middle school	0.350***	0.103	0.096	0.188***	0.104	0.049	0.629**	0.882***	0.435***	0.128*
	(0.073)	(0.086)	(0.079)	(0.071)	(0.120)	(0.101)	(0.275)	(0.257)	(0.108)	(0.067)
High school and above	0.778***	0.471***	-0.337**	0.418***	0.143	0.252	0.885	1.550***	0.784***	0.237**
	(0.124)	(0.143)	(0.145)	(0.127)	(0.246)	(0.201)	(0.566)	(0.526)	(0.152)	(0.101)
Single (Refs: Married)	-0.147**	-0.089	0.118	-0.097	-6.576***	-7.283***	-2.661***	-3.202***	0.020	-0.095
	(0.073)	(0.086)	(0.078)	(0.070)	(0.367)	(0.358)	(0.294)	(0.277)	(0.098)	(0.065)
Constant	-1.594*	-2.340***	-3.290***	0.321	7.101***	9.695***	-10.962	-7.456	10.275	-1.067
	(0.814)	(0.891)	(1.005)	(1.106)	(2.730)	(2.558)	(5729.691)	(5729.690)	(620.520)	(1.120)
pseudo-R-squared	0.04	0.02	0.02	0.03	0.39		0.09		0.13	
OBS	10130	10129	10129	10129	9767		9767		10716	

Data Source: CHARLS, 2018.

Notes: 1) binomial logistic regression is used for binominal dependent variable while multinomial (polytomous) logistic regression is used for multinominal dependent variable; 2) standard errors (robust) in parentheses, sig:

* p<0.05

** p<0.01

*** p<0.001.

## Discussion

Our analysis found that compared with the non-empty nest elderly, the empty nest elderly had higher proportion of dyslipidemia, diabetes, chronic lung diseases and heart attack. Previous studies have also shown that compared with non-empty nest elderly, empty nest elderly have a higher risk of endocrine disorders and immune disorders [26,27]. As suggested by our study, coupled with unhealthy lifestyle and lack of care from their children, empty nest elderly may be more likely to suffer from diseases such as diabetes and dyslipidemia.

Moreover, our analysis showed that empty nest elderly had higher proportion of participants who were "married and lived with spouse” as compared to non-empty nest elderly. While further research is needed for this interesting finding, we found that empty nest elderly had higher proportion of participants who felt satisfied with their marriage than non-empty nest elderly. Similarly, a previous study found that empty nest elderly who lived as couples were more likely to be satisfied with their marriage and had the lower depression scores than non-empty nest elderly who lived as couples [28]. This finding might be related to the intergenerational dynamics experienced by non-empty nest elderly: a significant proportion of elders who currently live with adult children have to undertake care duties for their grandchildren and thus have relatively less time left to interact with their spouse [29]. By contrast, among empty nest elderly, as the spouse have sufficient time to interact among themselves, they may experience higher sense of satisfaction about their marriage. Contrary to the previous study in China [30], the empty nest elderly had higher proportion of participants who felt satisfied with the children’s relationship than non-empty nest elderly. Traditional filial piety has shaped older people’s expectation that their adult children are responsible to provide all forms of support in old age. However, when their children leave home, empty nest elderly may have more time to organize their own lives than non-empty nest elderly, may avoid some of the contradictions and conflicts encountered in co-living with their children, and may have different feelings in their relationship with their children [31].

Our analysis also showed that, consistent with other studies [32], the lifestyle of empty nest elderly was relatively unhealthy, as empty nest elderly had higher proportion of participants who drank more frequently than non-empty nest elderly. Non-empty nest elderly tended to have better lifestyles perhaps because they had more opportunities to get along with their children, who for example, could advise on lifestyle behavior in a timely manner.

Unexpectedly, our multivariable analysis showed that no statistical difference was found between empty nest elderly and non-empty nest elderly regarding dyslipidemia, diabetes, chronic lung diseases, heart attack, marriage satisfaction, children satisfaction and frequency of alcoholic drinking. Indeed, research of Chinese families and intergenerational relations have identified a new form of intergenerational living since the 1990s, that is, live separately but close by. Scholars have coined terms such as “aggregate family” [33] or “networked family” to refer to this new form of intergenerational living; living close by is the most preferred form of living among the ageing population in China. While the survey data did not allow us to investigate this form of living and its impact upon the health and well-being of ageing parents, we speculated that the no differences in health status and well-being between non-empty nest elderly and empty nest elderly might be related to the fact that neither form of living is ideal for the ageing parents. However, the majority of our study participants, both empty and non-empty nest elderly, were rural (66.9%). Previous studies have also shown that adult children in rural areas do not usually move too far away from where their parents live and generally move to nearby villages [3], so they can easily meet up and support each other. Also, the participants chosen for our study were born before 1958, a generation that, because of the absence of the "one-child policy", often had many siblings and tended to live together. Their health and wellbeing (e.g., loneliness) of the empty-nest elderly due to the absence of their adult children could be compensated for by convenient support from their siblings. Therefore, this generation of empty nest elderly is not completely "empty" and their health and well-being may not differ significantly from that of non-empty nest elderly.

More importantly, China have been actively addressing population ageing, which may help to reduce the gap in health or well-being between empty nest elderly and non-empty nest elderly. Thanks to the rapid economic growth, China has improved the life expectancy and educational attainment, and reduced disability among the elderly. It has also improved the health care system for the elderly, e.g., through expansion of access to public health services in both urban and rural areas [34]. Various models of integrated care are being explored and implemented to adapt to the development of the aging population and improve the health status and quality of life for the elderly [35].

Although we do not find significant difference with regards to the study variables of health and wellbeing among the empty nest and non-empty nest elderly in the multivariable analysis, a few policy implications can be drawn from this study:

Firstly, increased attention and guidance should be given to the physical and mental health problems of empty nest elderly, through more proactive health promotion and intervention among the empty nest elderly, as our Chi-square analysis suggested higher proportion of participants suffering from dyslipidemia, diabetes, chronic lung diseases and heart attack among empty nest elderly than that of non-empty nest elderly.

Secondly, empty nest elderly should still be given more family and social support. Contrary to our cognition, our study found empty nest elderly had higher proportion of participants who were satisfied with life, health, marriage, children than non-empty nest elderly. While this implies the need to strengthen the family and social support for non-empty elderly, equally important support should also be given to the empty nest elderly since inconsistent results were identified from different studies [14,36]. For instance, it is necessary to provide education and training on how to improve communication among couples and between elderly and their children, among both empty nest and non-empty nest elderly.

Thirdly, it is important to improve the supply of aged care services among empty nest elderly since we found that empty nest elderly had a worse lifestyle than non-empty nest elderly in univariate analysis. With population aging in China, it is also important to develop community or home- based elderly care system to meet the increasing demand of elderly. Community should be targeted as an important platform where information and suggestions on healthy ageing are communicated to the elders to improve their lifestyles and social activities [37]. In other words, community could serve as an important bridge to offset the distance in health status between empty nest elderly and non-empty nest elderly.

### Limitations

There are several limitations of this study. First, there was no authoritative and unified definitions and standards on the empty nest elderly. Due to limitation of the survey data and using second-hand data for the research, we could not take into consideration the distance between ageing parents and their adult children who lived close by. It would be interesting to evaluate whether this compromise between empty nest elderly and non-empty nest elderly have produced better consequences on health and wellbeing for the ageing parents. Second, although this study carried on the comparative analysis from the comprehensive aspect, it cannot consider all aspects, such as economic income. Third, we only divided the participants into empty nest and non-empty nest, and future research can consider different subgroups under both groups (such as ages, number of children, living alone or with spouse, living in rural or urban areas), to improve the robustness of the comparation. Finally, 324 participants (i.e., 2.9% of the total samples) with age missing were removed from our analysis; furthermore, missing values existed among other variables, but this only remained between 0.5% and 10%, thus resulting in little bias in a large-sample analysis. Despite these limitations, results from this study will provide reference for promoting healthy aging, especially among empty nest elderly.

## Conclusions

Our study identified significant between-group differences of the health and wellbeing among the empty nest elderly and non-empty nest elderly regarding the development of dyslipidemia, diabetes, chronic lung diseases, heart attack, frequency of drinking, satisfaction with relationships with spouses and children. However, disappearance of such difference in the multivariable analysis may indicate improved health and wellbeing among the empty elderly. Even though our study still suggested the importance of improving the health, lifestyles, family dynamics of the elderly and promoting the integration of health and social care for the elderly, especially among the empty-nest elderly.

## Supporting information

S1 Checklist STROBE statement—checklist of items that should be included in reports of observational studies. (DOCX)

S1 File Study’s minimal underlying data set. (ZIP)

S2 File The weighted process of Table 1. (ZIP)

S3 File The weighted process of Table 2. (ZIP)

S4 File The weighted process of Table 3. (ZIP)

S5 File The weighted process of Table 4. (ZIP)
