# Supplementary material for: Health and wellbeing among the empty nest and non-empty nest elderly in China—Results from a national cross-sectional study
Source: PLoS One. 2023 Sep 12;18(9):e0291231. doi: 10.1371/journal.pone.0291231 (PMC10497119; doi:10.1371/journal.pone.0291231)
Supplement: S1 Checklist — (DOCX) [file pone.0291231.s001.docx]

STROBE Statement—checklist of items that should be included in reports of observational studies

|  | Item No. | Recommendation | Page  No. | Relevant text from manuscript |
| --- | --- | --- | --- | --- |
| **Title and abstract** | 1 | (*a*) Indicate the study’s design with a commonly used term in the title or the abstract | 1 | Results from a National Cross-sectional Study |
|  |  | (*b*) Provide in the abstract an informative and balanced summary of what was done and what was found | 2 | This study reported the health and well-being of empty nest elderly with regards to their health status, depression and satisfaction, lifestyle as compared to non-empty nest elderly in China. |
| Introduction | | | |  |
| Background/rationale | 2 | Explain the scientific background and rationale for the investigation being reported | 4-5 | The number of empty nest elderly in China has gradually increased in recent years. There is growing concern about the physical and mental health of this population as empty nest elderly are commonly at the risk of compromising health, home safety and quality of life. |
| Objectives | 3 | State specific objectives, including any prespecified hypotheses | 5 | this study aims to compare the health and well-being between empty nest elderly and non-empty nest elderly, with regards to the demographic factors, health status, depression, satisfaction and lifestyle |
| Methods | | | |  |
| Study design | 4 | Present key elements of study design early in the paper | 7 | Key Variable Definitions  Empty Nest Elderly |
| Setting | 5 | Describe the setting, locations, and relevant dates, including periods of recruitment, exposure, follow-up, and data collection | 5-6 | The data used in this paper was exported from the national survey of China Health and Retirement Longitudinal Survey in 2018 (CHARLS2018).  The CHARLS national baseline survey was conducted in 28 provinces, 150 countries/ districts, 450 villages/ urban communities across the country in 2011-2012, with wave 2, wave 3 and wave 4 conducted in 2013, 2015 and 2018 respectively. |
| Participants | 6 | (*a*) *Cohort study*—Give the eligibility criteria, and the sources and methods of selection of participants. Describe methods of follow-up  *Case-control study*—Give the eligibility criteria, and the sources and methods of case ascertainment and control selection. Give the rationale for the choice of cases and controls  *Cross-sectional study*—Give the eligibility criteria, and the sources and methods of selection of participants | 6 | We created separate weights for households, individuals, and biomarkers to deal with nonresponse bias and sampling-frame errors. After merging variables from the sub-databases and constructing the weights, 236 participants were excluded. Finally, a total of 10,818 valid samples were included in our study |
|  |  | (*b*) *Cohort study*—For matched studies, give matching criteria and number of exposed and unexposed  *Case-control study*—For matched studies, give matching criteria and the number of controls per case | NA |  |
| Variables | 7 | Clearly define all outcomes, exposures, predictors, potential confounders, and effect modifiers. Give diagnostic criteria, if applicable | 7-8 | empty nest elderly are defined as those aged 60 years or older, having no children or living alone without their children for 12 months  The socio-demographic variables included age, gender, number of children, type of residence, location of residential address, education and marital status. |
| Data sources/ measurement | 8* | For each variable of interest, give sources of data and details of methods of assessment (measurement). Describe comparability of assessment methods if there is more than one group | *8* | *Health status variables included self-assessed health status, diagnosed with hypertension, diagnosed with dyslipidemia, diagnosed with diabetes, diagnosed with chronic lung diseases, diagnosed with heart attack, diagnosed with stroke, diagnosed with emotional, nervous or psychiatric problems. Depression and satisfaction variables included have depressive symptoms, life satisfaction, health satisfaction, marital satisfaction and satisfaction with children's relationship. Depression is measured based on the CES-D10 scale, which covers 10 questions in the CHARLS2018 questionnaire. Participants with total score ≥10 and <10 are judged as having or not having depressive symptoms. Lifestyle variables included average sleep, vigorous-intensity physical activities, moderate physical activities, mild physical activities, social activities, ever smoked, frequency of drinking.* |
| Bias | 9 | Describe any efforts to address potential sources of bias | 25 | Finally, 236 participants with missing values were removed from our analysis; however, this only accounted for 2.13%, of the total samples, resulting in little bias. |
| Study size | 10 | Explain how the study size was arrived at | 6 | We created separate weights for households, individuals, and biomarkers to deal with nonresponse bias and sampling-frame errors. After merging variables from the sub-databases and constructing the weights, 236 participants were excluded. Finally, a total of 10,818 valid samples were included in our study, covering 4,630 empty nest elderly and 6,188 non-empty nest elderly. |

Continued on next page

| Quantitative variables | 11 | Explain how quantitative variables were handled in the analyses. If applicable, describe which groupings were chosen and why | 8 | The variable of empty nesting or not was obtained by reconstructing the “Number of Children” variable and combining it with the “Where is [XChildName[𝑖]] Living Regularly” question and the “How Long Live with XChildName[𝑖]” question - where the number of children for non-empty nest elderly was not “0”; in the “Where is [XChildName[𝑖]] Living Regularly” question, those who showed “living with family respondent” were non-empty nest elderly, otherwise were empty nest elderly; in the “How Long Live with XChildName[𝑖]” question, those who showed “0 (months)” were empty nest elderly, otherwise were non-empty nest elderly. |
| --- | --- | --- | --- | --- |
| Statistical methods | 12 | (*a*) Describe all statistical methods, including those used to control for confounding | 9 | The socio-demographic variables (including number of children, age, gender, type of residential address, location of residential address, highest level of education and marital status variables) were used as control variables in logistic regression analysis. |
|  |  | (*b*) Describe any methods used to examine subgroups and interactions | 8 | Chi-square test was used to analyze and compare the differences between these two groups in these variables. Bivariate Logistic Regression and Multivariate Logistic Regression were employed to confirm whether the status of empty nesting was associated with the health status, depression, satisfaction, lifestyle and other variables of the elderly. |
|  |  | (*c*) Explain how missing data were addressed | 6 | After merging variables from the sub-databases and constructing the weights, 236 participants were excluded. |
|  |  | (*d*) *Cohort study*—If applicable, explain how loss to follow-up was addressed  *Case-control study*—If applicable, explain how matching of cases and controls was addressed  *Cross-sectional study*—If applicable, describe analytical methods taking account of sampling strategy | NA |  |
|  |  | (*e*) Describe any sensitivity analyses | NA |  |
| Results | | | | |
| Participants | 13* | (a) Report numbers of individuals at each stage of study—eg numbers potentially eligible, examined for eligibility, confirmed eligible, included in the study, completing follow-up, and analysed | 6 | We created separate weights for households, individuals, and biomarkers to deal with nonresponse bias and sampling-frame errors. After merging variables from the sub-databases and constructing the weights, 236 participants were excluded. Finally, a total of 10,818 valid samples were included in our study, covering 4,630 empty nest elderly and 6,188 non-empty nest elderly. |
|  |  | (b) Give reasons for non-participation at each stage | 6 | After merging variables from the sub-databases and constructing the weights, 236 participants were excluded. |
|  |  | (c) Consider use of a flow diagram | 6 | Fig 1. Flow chart of study sample selection. |
| Descriptive data | 14* | (a) Give characteristics of study participants (eg demographic, clinical, social) and information on exposures and potential confounders | 8 | The socio-demographic variables included age, gender, number of children, type of residence, location of residential address, education and marital status. |
|  |  | (b) Indicate number of participants with missing data for each variable of interest | 6 | 236 participants were excluded |
|  |  | (c) *Cohort study*—Summarise follow-up time (eg, average and total amount) | NA |  |
| Outcome data | 15* | *Cohort study*—Report numbers of outcome events or summary measures over time | *NA* |  |
|  |  | *Case-control study—*Report numbers in each exposure category, or summary measures of exposure | *NA* |  |
|  |  | *Cross-sectional study—*Report numbers of outcome events or summary measures | *10* | *Of 10,818 elderly people, 4,630 (42.8%) and 6,188 (57.2%) were empty nest elderly and non-empty nest elderly respectively.* |
| Main results | 16 | (*a*) Give unadjusted estimates and, if applicable, confounder-adjusted estimates and their precision (eg, 95% confidence interval). Make clear which confounders were adjusted for and why they were included | 17-20 | Table 5. Regression Analysis for Full Sample |
|  |  | (*b*) Report category boundaries when continuous variables were categorized | 10, 11, 16 | Age  Number of Children  Average Sleep Time |
|  |  | (*c*) If relevant, consider translating estimates of relative risk into absolute risk for a meaningful time period | NA |  |

Continued on next page

| Other analyses | 17 | Report other analyses done—eg analyses of subgroups and interactions, and sensitivity analyses | NA |  |
| --- | --- | --- | --- | --- |
| Discussion | | | | |
| Key results | 18 | Summarise key results with reference to study objectives | 26 | Our study showed that the health and well-being of empty nest elderly tended to be worse than that of non-empty nest elderly, although no significant difference was found regarding the development of dyslipidemia, diabetes, emotional problems and some lifestyle problems among both groups. |
| Limitations | 19 | Discuss limitations of the study, taking into account sources of potential bias or imprecision. Discuss both direction and magnitude of any potential bias | 25 | no authoritative and unified definitions and standards on the empty nest elderly;  it cannot consider all aspects;  we only divided the participants into empty nest and non-empty nest |
| Interpretation | 20 | Give a cautious overall interpretation of results considering objectives, limitations, multiplicity of analyses, results from similar studies, and other relevant evidence | 20-23 | Previous studies have also shown that compared with non-empty nest elderly, empty nest elderly have a higher risk of endocrine disorders and immune disorders;  This finding might be related to the intergenerational dynamics experienced by non-empty nest elderly;  research of Chinese families and intergenerational relations have identified a new form of intergenerational living since the 1990s;  This may have reduced the gap in health or well-being between empty nest elderly and non-empty nest elderly |
| Generalisability | 21 | Discuss the generalisability (external validity) of the study results | 23-25 | increased attention and guidance should be given to the physical and mental health problems of empty nest elderly;  empty nest elderly should be given more family and social support as our univariate analysis found that the satisfaction of empty nest elderly with their children was lower;  it is important to improve the supply of aged care services among empty nest elderly since we found that empty nest elderly had a worse lifestyle |
| Other information | |  | | |
| Funding | 22 | Give the source of funding and the role of the funders for the present study and, if applicable, for the original study on which the present article is based | 27 | This work was supported by the National Social Science Foundation of China (Grant Number 20&ZD122) and the Social Science Foundation of Guangdong Province (Grant Number GD20CGL32). |

*Give information separately for cases and controls in case-control studies and, if applicable, for exposed and unexposed groups in cohort and cross-sectional studies.

**Note:** An Explanation and Elaboration article discusses each checklist item and gives methodological background and published examples of transparent reporting. The STROBE checklist is best used in conjunction with this article (freely available on the Web sites of PLoS Medicine at http://www.plosmedicine.org/, Annals of Internal Medicine at http://www.annals.org/, and Epidemiology at http://www.epidem.com/). Information on the STROBE Initiative is available at www.strobe-statement.org.
